# Induced Ketosis as a Treatment for Neuroprogressive Disorders: Food for Thought?

**DOI:** 10.1093/ijnp/pyaa008

**Published:** 2020-02-08

**Authors:** Gerwyn Morris, Basant K Puri, Andre Carvalho, Michael Maes, Michael Berk, Anu Ruusunen, Lisa Olive

**Affiliations:** 1 The Institute for Mental and Physical Health and Clinical Translation, School of Medicine, Deakin University, Australia; 2 C.A.R., Cambridge, United Kingdom; 3 Hammersmith Hospital, London, United Kingdom; 4 Centre for Addiction and Mental Health (CAMH), Toronto, ON, Canada; 5 Department of Psychiatry, University of Toronto, Toronto, ON, Canada; 6 Department of Psychiatry and Medical Psychology, Medical Faculty, Medical University of Plovdiv, Plovdiv, Bulgaria; 7 Department of Psychiatry, Faculty of Medicine, Chulalongkorn University, Bangkok, Thailand; 8 Orygen, The National Centre of Excellence in Youth Mental Health, the Department of Psychiatry, and the Florey Institute for Neuroscience and Mental Health, University of Melbourne, Australia

## Abstract

Induced ketosis (or ketone body ingestion) can ameliorate several changes associated with neuroprogressive disorders, including schizophrenia, bipolar disorder, and major depressive disorder. Thus, the effects of glucose hypometabolism can be bypassed through the entry of beta-hydroxybutyrate, providing an alternative source of energy to glucose. The weight of evidence suggests that induced ketosis reduces levels of oxidative stress, mitochondrial dysfunction, and inflammation—core features of the above disorders. There are also data to suggest that induced ketosis may be able to target other molecules and signaling pathways whose levels and/or activity are also known to be abnormal in at least some patients suffering from these illnesses such as peroxisome proliferator-activated receptors, increased activity of the Kelch-like ECH-associated protein/nuclear factor erythroid 2-related factor 2, Sirtuin-1 nuclear factor-κB p65, and nicotinamide adenine dinucleotide (NAD). This review explains the mechanisms by which induced ketosis might reduce mitochondrial dysfunction, inflammation, and oxidative stress in neuropsychiatric disorders and ameliorate abnormal levels of molecules and signaling pathways that also appear to contribute to the pathophysiology of these illnesses. This review also examines safety data relating to induced ketosis over the long term and discusses the design of future studies.

## Introduction

**Figure 1.**
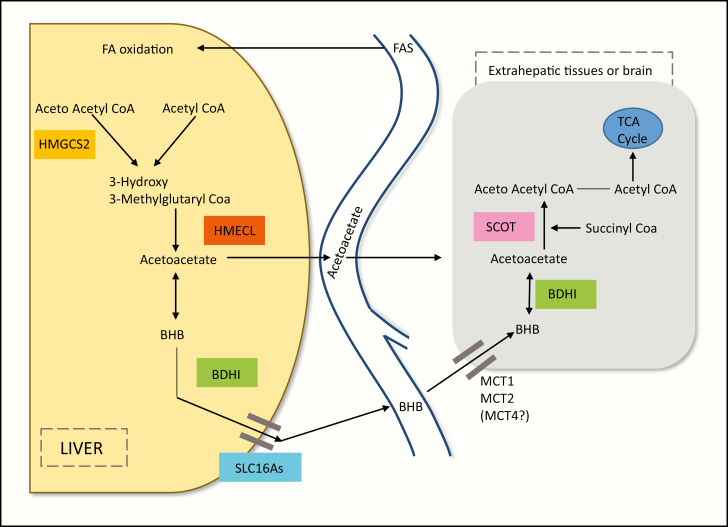
The biochemistry of ketogenesis. Prolonged glucose restriction leads to an increased glucagon insulin ratio, relieving the inhibition of adipose triglyceride lipase and hormone-sensitive lipase, which are key enzymes in the production of free fatty acids (FFAs) in adipocytes and their subsequent release into the peripheral circulation. Decreased levels of insulin and glucose also combine to relieve the inhibition of carnitine acyltransferase 1 in the liver, which governs the uptake of FFAs into mitochondria, and to increase levels of 3-hydroxy-3-methyl-glutaryl-coenzyme A (HMG-CoA) while reducing levels of oxaloacetate, as it is used as a precursor for the manufacture of glucose. The net effect of these changes is increased glucagon-mediated transport of FFAs into the liver and increased uptake into mitochondria where they are used for the manufacture of acetyl coenzyme A (acetyle-CoA). In normal conditions, this would enter the tricarboxylic acid (TCA) cycle, but in an environment of reduced oxaloacetate the only metabolic pathway open to the molecule is ketogenesis involving the formation of ketone bodies via a series of steps. In the first of these reactions, Acetyl-CoA (AcCoA) is converted to acetoacetyl CoA in a reaction enabled by 3-Ketothiolase. This molecular intermediate is then converted to HMG CoA by HMG-CoA synthase, which is constitutively expressed in mitochondria. The final reaction in this pathway is the cleavage of HMG-CoA by HMG-CoA Lyase to produce acetoacetate. beta-hydroxybutyrate (BHB) may then be formed by the reversible reduction of acetoacetate mediated by 3-hydroxybutyrate dehydrogenase, and acetone may be produced by the thermodynamically favorable decarboxylation of aceoaetic acid (AA). Egress of these ketone bodies from the liver is facilitated by the transporter Solute Carrier Family 16, Member 6 (SLC16A6). Their subsequent entry into peripheral tissues and brain facilitated by monocarboxylic acid transporters ultimately serves as a source of AcCoA for the TCA cycle. Once in situ, BHB may be reconverted to acetoacetate in a reaction enabled by the same enzyme. However, from that point, ketolysis and utilization of ketone bodies display major biochemical differences compared with ketogenesis. In particular, Succinyl-CoA transfers its CoA group to acetoacetate to produce acetoacetyl-CoA in a reaction enabled by the enzyme succinyl-CoA:3-ketoacid coenzyme A transferase (also known as OXCT1 or SCOT), bypassing the irreversible step in ketogenesis catalyzed by HMG-CoA synthase and thereby preventing the development of a futile cycle of hepatic BHB synthesis and utilization.

Diet-induced ketosis and/or ingestion of ketone bodies (KBs) is an established treatment for children (Neal et al., 2008, 2009) and adults with pharmacologically resistant epilepsy (Klein et al., 2014; Liu et al., 2018). Research teams have reported some success in ameliorating the severity of symptoms in neurodegenerative diseases, most notably in patients with mild cognitive impairment or early Alzheimer’s disease (for review, see Lange et al., 2017), more recently Parkinson’s disease (Phillips et al., 2018), and autistic spectrum disorders (for review, see Elamin et al., 2017). There is also some, albeit limited, evidence that nutritional ketosis may reduce symptoms in some patients with schizophrenia (SZ) (Włodarczyk et al., 2018), bipolar disorder (BPD) (Phelps et al., 2013), and major depressive disorder (MDD) (Brietzke et al., 2018) (Bostock et al., 2017a). It is worthy of note that MDD, BPD, and SZ are being increasingly described as neuroprogressive disorders to reflect progressive neuroanatomical and cognitive decline driven by many common factors present in each illness such as inflammation in the periphery and the brain, nitroxidative stress mitochondrial dysfunction coupled with disrupted tryptophan metabolism, and deficiencies in glutamatergic neurotransmission and neurotropin activity and increased production of cortisone coupled with impaired performance of glucocorticoid receptors (Berk et al., 2011; Maes et al., 2011; Davis et al., 2014; Haroon and Miller, 2017). The presence of these abnormalities in each neuroprogressive illness is unsurprising as evidence suggests that they may be the result of mitochondrial dysfunction oxidative stress and mitochondrial dysfunction in the periphery and in the brain (Morris et al., 2015a, 2017, 2019b). This may be of considerable clinical relevance as evidence suggests that many of the effects of induced ketosis would appear to be desirable as far as the treatment of these illnesses is concerned. Readers interested in the biochemistry underpinning the development of induced ketosis are invited to consult Figure 1.

For example, there is accumulating preclinical and clinical evidence that dietary ketosis results in the amelioration of oxidative stress, mitochondrial dysfunction, and inflammation in the periphery and in the brain of animals and humans (Jarrett et al., 2008; Nylen et al., 2009; Milder et al., 2010; Milder and Patel, 2012; Kim et al., 2015; Greco et al., 2016; Hasan-Olive et al., 2019). This could provide a possible approach that might reduce the magnitude of inflammation oxidative stress and mitochondrial dysfunction, which may be the core drivers of many of the symptoms associated with neuroprogressive disorders (Morris and Berk, 2015; Morris et al., 2015b).

Prolonged ingestion of a ketogenic diet (KD) or the ketone body, beta-hydroxybutyrate (BHB) leads to a global upregulation of peroxisome proliferator-activated receptors (PPARs) and increased activity of the Kelch-like ECH-associated protein-1/Nrf-2 system throughout the brain, at least as far as rodent data are concerned (Jeong et al., 2011; Simeone et al., 2017a; Knowles et al., 2018). Ingestion of a KD or BHB also decreases levels of nuclear factor (NF)-κB p65, most notably in microglia (Fu et al., 2014, 2015; Harun-Or-Rashid and Inman, 2018). There is a wealth of in vivo data associating the entry of KBs into glial cells and neurons, facilitated by monocarboxylate transporters, with the upregulation of several transcription factors, cofactors, and enzymes such as Sirtuin-1 (SIRT-1), SIRT-3, and Peroxisome proliferator-activated receptor-gamma coactivator (PGC)-1-alpha (Scheibye-Knudsen et al., 2014; McCarty et al., 2015; Elamin et al., 2018b; Hasan-Olive et al., 2019), which play a major role in regulating energy production, multiple aspects of cellular metabolism, and cellular redox status (Gano et al., 2014; Newman and Verdin, 2014; Veech et al., 2017; Miller et al., 2018).

These results offer the potential for ameliorating the effects of other likely sources of pathology in neuroprogressive disorders such as the inhibition of the Nrf-2 system and the compensatory antioxidant response (Genc and Genc, 2009; Martin-Hernandez et al., 2018; Zhang et al., 2018; Morris et al., 2019d). Elevated NF-KB activity is also thought to be a factor involved in driving the high levels of neuroinflammation, which in turn, is thought to play a major role in the pathophysiology, and possibly the pathogenesis, of neuroprogressive disorders (Thibaut, 2017; for review, see Kopitar-Jerala, 2015). Similarly, several research teams have reported decreased PPARγ activity and/or levels in the brains of individuals diagnosed with BPD (Nierenberg et al., 2018) and first-episode SZ (García-Bueno et al., 2014). This may also be of pathophysiological importance as it suggests impaired fatty acid oxidation and a failure to adjust energy supply in the face of changing metabolic environments (Poulsen et al., 2012; Chowdhury et al., 2018). This may be of paramount significance as fatty acid oxidation is a vital process enabling the maintenance of brain function and neural survival in an environment of glucose hypometabolism observed in SZ (Seethalakshmi et al., 2006), BPD (Fabrazzo, 2018), and MDD (Su et al., 2014) and is increasingly considered to be a factor in the pathogenesis and pathophysiology of these illnesses. Dysregulated or reduced SIRT-1 signaling also appears to be a characteristic feature of neuroprogressive diseases (Kishi et al., 2011; Nivoli et al., 2016; Lu et al., 2018; for review, see Alageel et al., 2018). The same appears to be true for PGC 1 alpha (for review, see Morris et al., 2013). These findings also suggest the presence of impaired energy metabolism and a failure of antioxidant systems in patients suffering from neuroprogressive disorders (Morris et al., 2019a).

The question arises as to the mechanisms underpinning these multidimensional, and potentially highly beneficial, effects that stem from induced ketosis or BHB administration, and the first objective of this paper aims to answer this question. In doing so, we will first focus on the properties of BHB as a free radical scavenger and activator of histone deacetylase (Shimazu et al., 2013; Kong et al., 2017; Wang et al., 2017) and then move on to discuss the plethora of favorable biochemical changes in antioxidant and bioenergetic profiles resulting from the upregulation of nicotinamide adenine dinucleotide+ (NAD+) that has been repeatedly observed in the brains of animals and in humans following protracted ketosis or ketonemia (Grabacka et al., 2016b; Elamin et al., 2017, 2018a; Xin et al., 2018). The physiological role of all the molecules discussed above is depicted in Table 1.

**Table 1.** Physiological Roles of Signalling Molecules Commonly Cited in the Body of the Paper

Molecules	Physiological role
NF-κB	Represents family of 5 structurally similar inducible transcription factors (p50, p52, RelA, RelB, and c-Rel) whose activity governs that of plethora of genes involved in effecting or regulating immune and inflammatory pathways and modulating several aspects of mitochondrial performance and energy production.
Nrf-2	A transcription factor that, once translocated to nucleus, associates with small Maf proteins and subsequently binds to ARE in promoter regions of target genes involved in cellular antioxidant response network, stimulating their transcription.
KEAP-1	A cysteine-rich molecule that binds to Nrf-2 in cytoplasm, promoting its degradation by ubiquitin proteasome pathway.
PPARα and PPARγ	Ligand-governed members of nuclear hormone receptor superfamily. Their activation generally increases expression of genes by binding to PPREs within their promoter regions in tandem with a retinoid X receptor. In certain circumstances, activation of PPARα or PPARγ may inhibit expression of gene clusters via interaction with other molecules such as NF-KB, SIRT-1, and PGC 1 alpha. PPARα and PPARγ activation provokes range of antioxidant, antiapoptotic, and antiinflammatory effects and plays major role in regulation of metabolism and mitochondrial dynamics.
FOXO	A FOXO class member of FOX protein family of transcription factors widely distributed in periphery and brain. Plays major role in regulating antioxidant responses, metabolism, energy production, and autophagy, including mitophagy and mitogenesis, by targeting promoter sequences on plethora of genes, generally leading to upregulation. This may be alone or in combination with range of other enzymes or coactivators such as SIRT-3, AMPK, and PGC 1alpha.
Sirtuins	Mammalian SIRTs function as NAD^+^-dependent deacylases and play many roles in regulating expression of genes involved in energy metabolism, cellular survival, inflammation, circadian rhythm regulation, and DNA repair. SIRT1 is found in cytosol and nucleus, modulates activity of transcription factors such as NF-KB p53, FOXOs, PPARs PGC1α, and PARP1. SIRT-3 is located in mitochondria and plays indispensable role in energy production and protecting organelles against oxidative and nitrosative stress.
Peroxisome proliferator-activated receptor-gamma coactivator	PGC-1alpha is a member of large family of transcription coactivators that acts as key player in regulation of energy metabolism by increasing mitochondrial biogenesis and stimulating mitochondrial respiration. Increased activity of this molecule also upregulates mitochondrial and cellular antioxidant responses.

Abbreviations: AMPK, AMP-activated protein kinase; ARE, antioxidant response element; FOX, forkhead box; KEAP1, Kelch ECH associating protein 1; NAD, Nicotinamide adenine dinucleotide; NF-κB, Nuclear factor-κB; Nrf-2, nuclear factor erythroid 2-related factor 2; PARP1, Poly (ADP-ribose) polymerase 1; PGC 1 alpha, Pparg coactivator 1 alpha; PPARalpha, Peroxisome proliferator-activated receptor alpha; PPRAgamma, Peroxisome proliferator-activated receptor gamma; PPREs, Peroxisome proliferator hormone response elements; SIRT-1, Sirtuin 1; SIRT-3, Sirtuin 3.

In addition, there is an increasing use of induced ketosis or BHB ingestion in the treatment of other illnesses other than epilepsy encouraged by several factors, including data from large prospective cohort studies suggesting that moderate or high carbohydrate intake is associated with significantly higher rates of mortality compared with diets low in carbohydrate content over an 8-year period (Dehghan et al., 2017). Such an increase in use has produced a large volume of efficacy and safety data following long-term administration in illnesses and conditions such as type 2 diabetes, metabolic syndrome, and obesity (Gupta et al., 2017). This is of particular importance when considering the long-term use of a KD or BHB supplementation in neuroprogressive disorders as these abnormalities are present in such patients at significantly higher levels than age- and sex-matched population norms (for review, see Morris et al., 2019c). Hence, reviewing the efficacy and safety evidence available from the long-term induction of ketosis and/or ketonemia in metabolic disorders and, indeed, epilepsy before arriving at a projection of the relative risks and benefits of each approach will be the second objective of this paper.

### Role of BHB in Free Radical Scavenging

Several research teams have produced in vivo data demonstrating that BHB and acetoacetate (ACA) administration can reduce oxidative stress by scavenging hydroxyl radicals and superoxide ions in various regions of the central nervous system (CNS), including the hippocampus and the neocortex. This results in reduced lipid peroxidation, improved ATP generation, and abrogation of glutamate excitotoxicity and synaptic dysfunction (Massieu et al., 2003; Maalouf et al., 2007; Haces et al., 2008; Julio-Amilpas et al., 2015). These findings have also been reported by research teams examining the effects of BHB and/or ACA on whole extracted neurons or CNS mitochondria in vitro (Maalouf et al., 2007; Haces et al., 2008; Maalouf and Rho, 2008; Julio-Amilpas et al., 2015). Reactive oxygen species (ROS) scavenging capacity also most likely underpins reports that KBs have the capacity to correct defective autophagy and ameliorate the effects of endoplasmic reticulum stress and the associated unfolded protein response (Camberos-Luna et al., 2016; Soejima et al., 2018).

### BHB and Upregulation of Uncoupling Proteins

Induced ketosis or BHB administration is associated with increased expression of a number of uncoupling proteins (UCPs), most notably UCP2, although UCP4 and UCP5 also appear to be upregulated, in the periphery and in the brain (Grav et al., 2003; Sullivan et al., 2004; Fisler and Warden, 2006; Malingriaux et al., 2013; Hasan-Olive et al., 2019). Increased activity of UCPs can diminish the mitochondrial membrane potential (ΔΨ), resulting in a decrease in ROS production. This has been associated with increased resistance to kainic acid-induced seizures (Kovac et al., 2012). Increased activity of UCPs leads to the uncoupling of oxidative phosphorylation and ATP production by allowing a partial dissipation of Δ*Ψ* or ΔpH by allowing the entry of protons from the inner membrane space at sites other than at ATP synthase (Kovac et al., 2012). This is of importance as Δ*Ψ* and Δ*p* are the drivers of electron transfer from complexes I, III, and IV to oxygen in the mitochondrial matrix (MM) (Brand et al., 2004) with the resulting formation of superoxide ions (Brand et al., 2004), and unsurprisingly the upregulation of UCPs is associated with a reduction in mitochondrial ROS (mtROS) production (Echtay, 2007; Mailloux and Harper, 2011). Given the decrease in Δ*Ψ* and Δ*p*, there is clearly a possibility that elevated UCP production could seriously compromise ATP production as discussed above. However, UCP elevation appears to be a very efficient defense against the advent of oxidative stress as a very modest dissipation of Δ*p* results in a large decrease in mtROS production and hence does not necessarily result in a significant decrease in the production of ATP (Votyakova and Reynolds, 2001; Lambert and Brand, 2004).

### Ketone Bodies (KBs) as Hydroxycarboxylic acid receptor 2 (HCA2) Ligands

In vivo data supplied by several research teams have established the role of BHB as a ligand for the G-protein-coupled HCA_2_ (Newman and Verdin, 2014; Guo et al., 2018; Trotta et al., 2019). BHB engagement with HCA_2_ has a major ameliorative effect on inflammation by inhibiting endoplasmic reticulum stress, resulting in decreased assembly of nucleotide-binding domain-like receptor protein 3 (Guo et al., 2018; Trotta et al., 2019). Details of the inhibitory effect of HCA_2_ activation on the suppression of inflammatory signaling in the periphery and brain may be obtained by reference to the work of (Graff et al., 2016).

The weight of evidence suggests that in vivo BHB engagement of HCA_2_ in activated microglia reduces activity of the pro-inflammatory enzymes Prostaglandin-endoperoxide synthase 2 (COX-2) and Inducible nitric oxide synthase (iNOS) by reducing degradation of NF of kappa light polypeptide gene enhancer in B-cells inhibitor, alpha and inhibiting the nuclear translocation of NF-κB, thereby preventing the action of the transcription factor in initiating and maintaining the transcription of proinflammatory cytokines and a range of other inflammatory molecules (Fu et al., 2014, 2015). Data also suggest that the downregulation of nucleotide-binding domain-like receptor protein 3 activity and decreased levels of Interleukin 1 beta (IL-1β) concomitant with reduced neuroinflammation seen in the CNS of study animals following BHB ingestion is mediated via HCA_2_ (Youm et al., 2015; Yamanashi et al., 2017; Trotta et al., 2019).

### BHB as A Class I and II Deacetylase Inhibitor

#### Background

**Figure 2.**
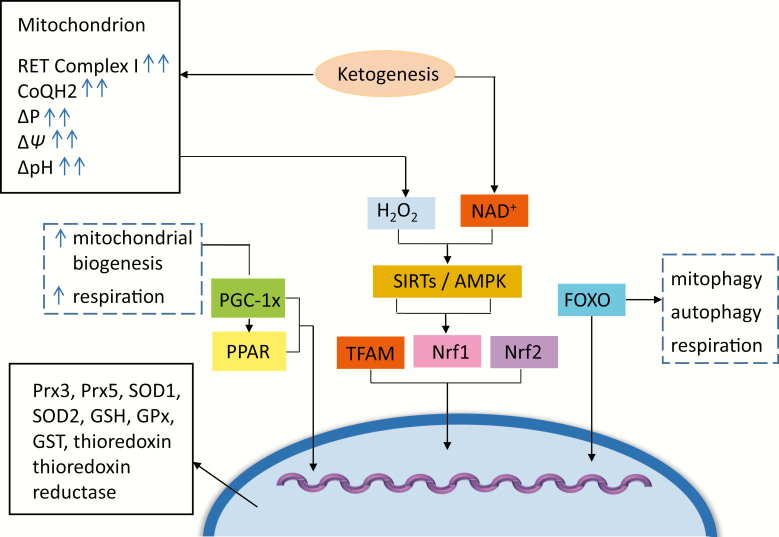
Mitochondrion. Glucose restriction and BHB oxidation leads to an increase in NAD+ and upregulated AMP-activated protein kinase (AMPK), with “downstream” activation of silent mating type information regulation 2 homologue 1 and 3 (SIRT1 and 3), peroxisome proliferator-activated receptor γ (PPARy), peroxisome proliferator-activated receptor γ coactivator 1α (PGC-1α), forkhead box O 3a (FOXO3a), and nuclear factor erythroid-derived 2-like 2 (NFE2L2). The cooperative activity of these enzymes and signalling systems ultimately result in increased transcription of genes related to oxidative capacity, mitochondrial uncoupling, and antioxidant defenses as detailed in the text. Fatty acid oxidation subsequent to ketolysis in an environment of glucose restriction decreases the ratio of NADH/FADH_2_, leading to the overreduction of the CoQ/CoQH2 couple due to an excess of electrons entering the ETC at complex II. This scenario may provoke increases in reverse electron transport and increased ROS production in the form of superoxide radicals at complex I of the ETC. The subsequent dismutation of superoxide to hydrogen peroxide in the mitochondrial matrix and “spill over” into the cytoplasm offer another mechanism whereby mitochondria to nuclear signaling activates a transcriptional response almost identical to the one initiated by glucose restriction and BHB formation described above. This response is likely to be a major player in increasing the cellular antioxidant response in stressed mitochondria characteristic of neuroprogressive disorders.

BHB acts as a class I and II deacetylase inhibitor that increases global levels of acetylation in vivo in a dose-dependent manner, resulting in the increased expression of specific genes involved in stimulating cellular antioxidant defenses and in an amelioration of oxidative stress (Shimazu et al., 2013; Kong et al., 2017; Wang et al., 2017). The weight of evidence suggests that such inhibition is associated with increased transcription and/or activity of metallothionein II, mitochondrial Superoxide Dismutase 2 (SOD2), catalase, Forkhead box O (FOXO) 3a, and Nrf2, (Shimazu et al., 2013; Wei et al., 2014; Nagao et al., 2016). Increased activity of FOXO3a and Nrf2 has significance as far as a global cellular antioxidant response is concerned, which we discuss below. These signaling pathways and the molecules involved are represented diagrammatically in Figure 2.

### Upregulation of Nrf2

Oxidative modification of selected cysteine thiol groups on Kelch-like ECH-associated protein (KEAP-1) is probably the most prevalent mechanism enabling dissociation of the Nrf2/KEAP-1 dimer in the cytosol enabling Nrf2 translocation to the nucleus (for review, see Morris et al., 2018). However, KEAP-1 dissociation and Nrf2 activation can also result from several covalent modifications of the latter, including phosphorylation and, in this case, acetylation (for review, see Ma, 2013).

Activation of Nrf2 leads to increased activity of gamma-glutamylcysteine ligase, which is the rate-limiting enzyme as far as glutathione synthesis is concerned, and the cysteine/glutamate antiporter (X_c_^−^), which ensures adequate levels of intracellular cysteine, the rate-limiting substrate for glutathione synthesis and reduces the efflux of oxidized glutathione into the extracellular environment, thereby preserving levels of glutathione within the cell (Steele et al., 2013; Ishii and Mann, 2014). Increased translocation of Nrf2 into the nucleus also leads to increased transcription of glutathione peroxidase, which plays an indispensable role in the reduction of highly toxic membrane lipid peroxides and maintenance of levels of hydrogen peroxide within physiological limits (Banning et al., 2005; Jablonska et al., 2015). Cytosolic mitochondrial and plasma membrane-bound and mitochondrial glutathione transferases are also upregulated by Nrf2 (Bartolini et al., 2015). These enzymes are important not least because they play an indispensable role in detoxifying electrophiles and phase 1-modified xenobiotics via conjugation prior to export into the extracellular environment (McWalter et al., 2004). These enzymes also exert other cytoprotective effects (Bartolini et al., 2015). Nrf2 also upregulates glutathione reductase activity, which provides another avenue for maintaining levels of glutathione (Harvey et al., 2009). Increased Nrf2 activity also upregulates the thioredoxin system increasing levels and activity of thioredoxin (TRX) and thioredoxin reductase 1 (Tanito et al., 2007; Im et al., 2012; Cebula et al., 2015). Nrf2, TRX, and thioredoxin reductase 1 also engage in complex self-sustaining, mutually reinforcing signaling with reductions in TRX1 activity leading to increasing Nrf2 activity and upregulated TRX activity increasing the transcriptional efficiency of Nrf2 (Li et al., 2016; Sueblinvong et al., 2016).

While there are many publications discussing the apex role of Nrf2 as the “master regulator” of cellular antioxidant defenses, the behavior of Nrf2 as an indispensable regulator of energy production is probably underdiscussed. There are accumulating data that this molecule also acts as an important regulator of mitochondrial structure and respiration via increasing fatty acid oxidation and ATP production via several different mechanisms following physical attachment to the outer mitochondrial membrane (Dinkova-Kostova and Abramov, 2015). Subsequent to such attachment, Nrf2 negatively regulates acetyl-CoA carboxylase, ATP-citrate lyase, stearoyl-CoA desaturase, and fatty acid synthase, which are all crucial enzymes enabling fatty acid synthesis. Importantly, a reduction in malonyl-CoA increases mitochondrial fatty acid oxidation, as this enzyme negatively regulates carnitine palmitoyltransferase 1 (Holmström et al., 2016). Nrf2 also plays a major role in maintaining the efficiency of the electron transfer chain (ETC) in an environment of chronic oxidative and nitrosative stress by stabilizing cytochrome *b*, cytochrome *c*, and cytochrome *c* oxidase (Venditti et al., 2009; Strom et al., 2016). The activity of this transcription factor also exerts a positive effect on mitochondrial dynamics by promoting association into networks and inducing mitophagy via mechanisms that are independent of mitochondrial membrane dissipation or the interplay between PTEN-induced putative kinase 1 and parkin (Holmström et al., 2016).

### Upregulation of FOXO3a

This transcription factor has a well-documented positive effect on the expression of a wide range of cellular antioxidant enzymes and other functional proteins, such as glutathione *S*-transferase, GPx1, GPx4, thioredoxin, thioredoxin reductases, peroxiredoxins (Prx) Prx3 and Prx5, selenoprotein P, metallothioneins I and II, and caeruloplasmin (Greer et al., 2007; Tan et al., 2008), as well as increasing the expression and activity of SOD2 and catalase (Greer et al., 2007; Li et al., 2009; Olmos et al., 2009; for review, see Klotz et al., 2015).

FOXO3a increases mitochondrial biogenesis and expression of transcription factor A, mitochondrial (Tseng et al., 2013). Furthermore, FOXO3a induces widespread mitochondrial gene expression (Peserico et al., 2013). This property is of importance as many such genes are involved in regulating mitochondrial mass, mitochondrial morphology, mitophagy, mitochondrial fusion and fission, and the production of ATP (Tseng et al., 2013; Zhou et al., 2017). While the effect on mitochondrial genes is broadly positive as far as improved organelle performance is concerned, somewhat counterintuitively, increased extra-mitochondrial FOXO3a activity may inhibit the activity of some nuclear genes involved in mitochondrial function, which could adversely affect ATP generation, at least in some circumstances (Ferber et al., 2012). However, the survival value of this action is emphasized by data highlighting a slight decrease in energy production but a reduction in mtROS production to a level below baseline (Ferber et al., 2012). The capacity of FOXO3a to regulate the balance between mtROS production and ATP production appears to be particularly important in stressed neurons and plays a major role in promoting their survival (Hagenbuchner and Ausserlechner, 2013).

### Consequences of BHB Oxidation, Decreased Glycolysis, and Increased NAD^+^ Levels

#### BHB and NAD+ “sparing”

Several research teams have reported an upregulation of NAD^+^ levels in the brains of their study animals and in humans following protracted ketosis (Grabacka et al., 2016b; Elamin et al., 2017, 2018a; Xin et al., 2018). Other authors have reported the inhibition or termination of glycolysis in the brains of human volunteers consuming a KD (Roy et al., 2012; Zhang et al., 2013b; for review, see Courchesne-Loyer et al., 2017a). These observations are interconnected and stem from relative differences in the number of NAD^+^ molecules reduced to NADH in the process of forming acetyl-CoA via glycolysis or BHB oxidation, as we explain below.

Briefly, the irreversible oxidation of glyceraldehyde-3 phosphatase to produce 3-phosphoglycerate, which is an indispensable step in the formation of pyruvate, is enabled by the action of glyceraldehyde-3 phosphate dehydrogenase. This enzyme requires NAD^+^ as a co-substrate leading to the reduction of 2 molecules of this cofactor to NADH per molecule of glyceraldehyde-3 phosphatase oxidized (Newman and Verdin, 2014; Elamin et al., 2018a). Once formed, pyruvate and NADH are translocated into the MM via the action of pyruvate translocase and several NAD^+^/NADH redox shuttles, for example, the malate-aspartate system, resulting in a relative depletion of the cytosolic NAD^+^ pool (Stein and Imai, 2012). Once in situ in the MM, NADH molecules are utilized as reducing equivalents for the tricarboxylic acid (TCA) cycle while each molecule of pyruvate is oxidized to 2 molecules of acetyl-CoA via the action of pyruvate dehydrogenase, whose action requires the reduction of 2 molecules of NAD^+^ per molecule of pyruvate oxidized. The oxidation of BHB to ACA and acetyl-CoA, on the other hand, is enabled by BHB dehydrogenase and succinyl-CoA:3 oxoacid (or ketoacid) CoA transferase and only requires the reduction of 2 molecules of NAD^+^ per 2 molecules of ACA formed as BHB dehydrogenase is the only NAD^+^-consuming enzyme in the process (Grabacka et al., 2016b; Puchalska and Crawford, 2017). This is clearly a very brief description of the biochemistry involved in glucose and BHB oxidation, but the key point to reemphasize is that the termination of glycolysis and increased oxidation of BHB subsequent to the advent of induced ketosis (Roy et al., 2012; Zhang et al., 2013b; Courchesne-Loyer et al., 2017a) effectively leads to an increased level of NAD^+^, which has a number of profound bioenergetic and metabolic consequences described in detail below (Elamin et al., 2018b).

### BHB and Increased NADH Oxidation in Mitochondria

There is also evidence to suggest that BHB increases NADH oxidation in mitochondria, leading to an increased NAD^+^/NADH ratio within organelles in the brain (Maalouf et al., 2007; Zhang et al., 2013a; Pawlosky et al., 2017). This would appear to be due, at least in part, to increases in complex I integrity and activity (Hughes et al., 2014; Frey et al., 2017; Paleologou et al., 2017). BHB also appears to stimulate the activity of succinate dehydrogenase (Tieu et al., 2003). An increased supply of succinate following its preferential oxidation over glucose may bypass complex I abnormalities by supplying electrons directly to complex II (Tieu et al., 2003; Sullivan et al., 2004). There is also some evidence to suggest that BHB may exert a direct and stimulatory effect on succinate dehydrogenase activity (Balietti et al., 2010). The potential benefit of BHB as the predominant source of electrons and reducing equivalents in an environment of ETC dysfunction or inhibition is further emphasized by data supplied by Kim and fellow workers, who demonstrated that complex II inhibition could be mitigated following BHB administration in the hippocampal neurons of their study animals (Kim et al., 2010). It would also appear that BHB oxidation increases the energetic status of electrons entering complex I and the redox difference between the NAD^+^/NADH couple and the CoQ/CoQH2 couple (Veech, 2004) with the effect of increasing the Gibbs free energy of hydrolysis of ATP molecules ultimately produced (Sato et al., 1995).

### Consequences of Increased NAD^+^/NADH Ratio

The increase in the NAD^+^/NADH ratio facilitated in the cytosolic and mitochondrial compartments following preferential BHB oxidation and/or increased BHB activity is of paramount importance. Increased NAD^+^ levels stimulate oxidative phosphorylation, ETC activity, and ATP production and affect multiple dimensions of cellular metabolism that govern adaptation in performance in response to different environmental conditions and changes in nutrient availability (Blacker and Duchen, 2016; Xiao et al., 2018; for review, see Stein and Imai, 2012). NAD^+^ may be phosphorylated in the cytosol and the NADP^+^-NADPH couple plays a major role in redox homeostasis in the periphery and the brain (Ying, 2008; Yang and Sauve, 2016; for review, see Xiao et al., 2018).

NAD^+^ levels and activity also modulate calcium homeostasis, regulate mitochondrial transition pore opening, and influence the transcription and posttranscriptional modification of hundreds of cellular proteins by activating PARP-1 and the sirtuin family of class III histone deacetylases, most notably the cytosolic SIRT-1 and the mitochondrial SIRT-3 (Kincaid and Bossy-Wetzel, 2013; Elamin et al., 2018a). Readers interested in more information regarding the location and mode of operation of individual sirtuins are referred to a comprehensive review on the subject by (Parodi-Rullán et al., 2018). It is important to note at the outset of this section that authors have reported the upregulation of SIRT-3 in the brains of their study animals following administration of a KD and/or BHB, which supports the data supplied by the authors above (Yin et al., 2015; Hasan-Olive et al., 2019). However, there is some suggestion that levels of SIRT-3 may be reduced in the periphery following BHB and/or KD intake in an environment where SIRT-1 (Srivastava et al., 2013) and SIRT-5 (Hutfles et al., 2017) are increased, for reasons that are not entirely clear (Srivastava et al., 2013). There is also some suggestion that SIRT-1 may be more sensitive than SIRT-3 to changes in NAD^+^ levels. However, we will discuss the actions of SIRT-3 before moving on to discuss the consequences of SIRT-1 activation as the activity of the former explains, at least in part, many of the apparent effects of BHB on the ETC and overall ATP production as detailed below.

### Increased Activity of SIRT-3

The activation of SIRT-3 has profound effects on the mitochondrial acetylome, affecting thousands of acyl groups on proteins performing essential functions in energy production and metabolic adaptation to a changing cellular environment. This leads to a global reprogramming of the mitochondrial proteome aimed at adapting to changes in energy demand, fuel supply, and redox status (Dittenhafer-Reed et al., 2015).

Many of the effects of BHB on the ETC and ATP production discussed above can be explained by the activation of SIRT-3 by elevated levels of NAD^+^ (Lombard et al., 2011). For example, SIRT-3 can bind to complexes I and II leading to an increase in their activity (Ahn et al., 2008; Cimen et al., 2010). The precise targets of SIRT-3 as far as complex I is concerned have not been fully elucidated, but direct binding to its NDUFA9 subunit appears to be one of the mechanisms involved (Ahn et al., 2008). Deacetylation/acetylation patterns of complex I subunits regulate basal ATP levels (Ahn et al., 2008). In terms of complex II, on the other hand, the stimulatory effect of SIRT-3 activation appear to be mediated by increased acetylation and activation of succinate dehydrogenase and succinate dehydrogenase flavoprotein (Cimen et al., 2010; Finley et al., 2011).

SIRT-3 also acts as the main regulator of fatty acid oxidation by reversible acetylation of long-chain acyl-CoA dehydrogenase (Hirschey et al., 2010a). This sirtuin also binds and deacetylates acetyl-CoA synthetase 2 (Hallows et al., 2006; Hirschey et al., 2010b) and thus plays an indispensable role in regulating the production of acetyl-CoA in the periphery and the CNS (Dittenhafer-Reed et al., 2015). Evidence suggests that this sirtuin exerts stimulatory effects on the TCA cycle by increasing the activity of glutamate dehydrogenase, resulting in enhanced metabolism of glutamate into α-ketoglutarate (Schlicker et al., 2008). SIRT-3 may also deacetylate isocitrate dehydrogenase 2, leading to an increase in the enzyme’s activity that also results in increased production of α-ketoglutarate (Yu et al., 2012; Sheng et al., 2015). Considered as a whole, the weight of evidence suggests that elevated SIRT-3 activity stimulates the TCA cycle by increasing the flux of carbon and the levels of metabolites that play an important role in regulating TCA cycle activity (Verdin et al., 2010; Finley and Haigis, 2012).

SIRT-3 also plays an important role in the regulation of mitochondrial redox homeostasis via increased acetylation and activity of mitochondrial SOD-2 (Kong et al., 2010; Zhang et al., 2016) and FOXO3a (Tseng et al., 2013; Rangarajan et al., 2015); the latter is capable of initiating a cascade of antioxidant enzymes and systems as detailed above. The cited study conducted by Rangarajan and fellow workers is of interest from the perspective of neuroprogressive diseases as the results demonstrate an increase in FOXO3a activity mediated by SIRT-3 in microglia in vivo (Rangarajan et al., 2015). Similarly, the study conducted by Zhang and others is of interest because the authors report an in vivo SIRT-3–mediated increase in FOXO3a activity in neurons (Zhang et al., 2016). There is also evidence to suggest that SIRT-3 activation may lead to the upregulation of glutathione peroxide, but it is not clear whether this is a direct effect or secondary to the activation of FOX03a (Kong et al., 2010). Readers interested in an in-depth treatment of the role of SIRT-3 in the maintenance of mitochondrial homeostasis are invited to consult an excellent review of the subject by (Kincaid and Bossy-Wetzel, 2013).

Finally, while SIRT-3 is highly sensitive to changes in NAD^+^ levels and thus a pivotal metabolic sensor, the enzyme may also be activated by several other mechanisms. For example, the transcription of SIRT-3 may be activated by the physical engagement of the alpha subunit of Nrf-2 or PGC-1α, so increased levels and activity of both transcription factors can also induce increased activity of mitochondrial SIRT-3 (Satterstrom et al., 2015; Zhang et al., 2016). In addition, several authors have reported that the antioxidant effects of SIRT-3 in the brain are mediated, at least in part, via the activation of PGC-1α (Zhang et al., 2016).

### Activation of SIRT-1

It should be noted that PGC-1α activity may also be increased in vivo by upregulation of SIRT-1, which is also activated by increased levels of NAD^+^ secondary to BHB oxidation so that the activation of SIRT-3 by PGC-1α is also dependent on ketolysis (Wang et al., 2015). Similarly, SIRT-1 may also acetylate and activate Nrf-2 and initiate a positive interaction between the 2 molecular entities, which plays a major reinforcing role in maintaining cellular antioxidant systems and fostering energy production in the face of increasing oxidative and nitrosative stress (Huang et al., 2017). Increased activity of SIRT-1 has been consistently reported in the brains of study animals following protracted ingestion of a KD or infusion of BHB (Scheibye-Knudsen et al., 2014; McCarty et al., 2015; Elamin et al., 2018b). There have also been reports of elevated SIRT-1 in the periphery under the same conditions (Srivastava et al., 2013).

Several authors have reported an association between SIRT-1 activation and an increase in mitochondrial biogenesis and beta-oxidation of fatty acids, with the latter facilitated via different mechanisms. These include increased fatty acid uptake into cells via the CD36 membrane transporter coupled with elevated influx of these molecules into mitochondria via upregulation of various carnitine palmitoyl transferase enzymes, including carnitine palmitoyltransferase 1 (Wanders et al., 2010; Liu et al., 2012; Vachharajani et al., 2014; for review, see Qu et al., 2016). Upregulated SIRT-1 activity also exerts several positive effects on mitochondria aimed at maximizing the efficiency of energy production. For example, SIRT-1 upregulation induces a signaling cascade of protein-protein interactions, ultimately aimed at terminating ATP generation via glycolysis and increasing ATP generation via fatty acid oxidation. Mechanisms involved in engineering this metabolic switch include suppression of lactate production (Elhanati et al., 2016; Long et al., 2017), downregulation of the glucose transporter 1, and inhibition of hypoxia-inducible factor 1-alpha signaling (Sebastian et al., 2012). SIRT-1 upregulation also offers a degree of mitochondrial protection in an environment of increasing oxidative and nitrosative stress. The primary mechanisms involved are the stimulation of cellular antioxidant defenses, via Nrf-2 activation, and promoting favorable mitochondrial dynamics such as increasing mitophagy, thereby removing damaged and dysfunctional mitochondria and maintaining mitochondrial membrane potential and so inhibiting the opening of the mitochondrial transition pore (Price et al., 2012b; Song et al., 2017).

### AMPK Activation

The weight of evidence suggests that levels and activity of adenosine monophosphate-activated protein kinase (AMPK) are increased in the brains of rodents following ingestion of a KD or BHB (Laeger et al., 2010; Genzer et al., 2015; Paoli et al., 2015). This also seems to be true of peripheral tissues (Kennedy et al., 2007; Badman et al., 2009). As previously discussed, evidence suggests that 1 route driving AMPK activation is increased activity of SIRT-1 (Price et al., 2012a). However, the relationship between SIRT-1 and AMPK is complex, and recent reports note an increase in SIRT-1 mRNA levels following AMPK activation (Dong et al., 2018). This may be due in part to the ability of AMPK to upregulate NAD^+^ by increasing levels of nicotinamide phosphoribosyl transferase (Fulco et al., 2008; Cantó et al., 2009). Furthermore, these molecular players seem to engage in a complex pattern of crosstalk with each molecule reinforcing and perpetuating the activity of the other and ultimately acting in partnership to regulate cellular bioenergetics and metabolism (for review, see Ruderman et al., 2010). The ability of AMPK to activate SIRT-1 is important because AMPK activation is part of the starvation response that is activated by the advent of dietary induced ketosis and increased levels of BHB (for review, see Newman and Verdin, 2014). Hence this mechanism may provide a route for the activation of SIRT-1 that is independent of BHB oxidation per se. SIRT-3 and AMPK can also engage in mutual activation and mutually reinforcing crosstalk aimed at regulating multiple dimensions of mitochondrial redox homeostasis and energy production (Chen et al., 2018; Zhao et al., 2018). Increased AMPK also increases the nuclear translocation of Nrf-2 (Joo et al., 2016) and would appear to increase its activity as a transcription factor, providing yet another route for increasing Nrf-2 activity stemming from induced ketosis (Mo et al., 2014).

### Upregulation of PGC-1α

While SIRT1, SIRT-3, and AMPK clearly have some direct effects on cellular energy production and mitochondrial survival, they also exert combined effects by deacylating and phosphorylating PGC-1α, leading to the activation of the latter molecule (Cantó et al., 2009; Kong et al., 2010). There is also some evidence to suggest that AMPK increases the expression of PGC-1α via a mechanism that remains to be delineated (Cantó et al., 2009). The activation of PGC-1α in turn positively modulates mitochondrial dynamics and increases mitochondrial biogenesis, oxidative phosphorylation, and oxygen consumption via a number of different mechanisms (Scarpulla, 2011; LeBleu et al., 2014).

One mechanism underpinning the positive effects of PGC-1α on energy production is the increased transcription of proteins involved in mitochondrial biogenesis and respiration (Valle et al., 2005; Lagouge et al., 2006). However, this protein also exerts a number of other beneficial effects on mitochondrial dynamics by inducing positive changes in the expression and activity of mitofusin-2 (MFN2), mitochondrial dynamin like GTPase (OPA1), dynamin related protein 1(Drp-1), and mitochondrial fission 1 protein (FIS-1) (Peng et al., 2017). The presence of data demonstrating the positive effects of PGC-1α in restoring the balance between mitochondrial fusion and fission in neurons also speaks to the potential merits of the KD as an adjunctive therapy in patients with neuroprogressive illnesses characterized by bioenergetic dysregulation (Dabrowska et al., 2015).

PGC-1α also increases the transcription of antioxidant proteins, including SOD1 (St-Pierre et al., 2006), SOD2 (St-Pierre et al., 2006), catalase (Valle et al., 2005), GPx (St-Pierre et al., 2003), thioredoxins (Valle et al., 2005), TRXR (Valle et al., 2005), Prx3 (Valle et al., 2005), and Prx5 (Valle et al., 2005) as well as the mitochondrial uncoupling proteins UCP2 (St-Pierre et al., 2003, 2006) and UCP3 (St-Pierre et al., 2003, 2006).

Once activated, PGC-1α interacts with the PPAR family of nuclear receptors and the FOXO family of transcription factors, thereby modulating their activity and location (Olmos et al., 2009; Wang, 2010) to influence expression of a variety of bioenergetic and antioxidant proteins (Puigserver and Spiegelman, 2003; Corona and Duchen, 2015). The combined effects of PGC-1α and PPARγ are mediated by complex formation (Olmos et al., 2013), and this physical interaction is pivotal in inducing the expression of a plethora of enzymes governing fatty acid metabolism and ketogenesis, which are characteristic of a KD (for review, see Grabacka et al., 2016a). Clearly, this route of FOXO activation differs from the increased acetylation induced by BHB directly acting as a deacetylase inhibitor, but the consequences are very much the same. Activated PGC-1α also increases expression of Nrf-2 via a mechanism dependent on p53, p38, and GSK3β (Aquilano et al., 2013; Choi et al., 2017). The activation and nuclear translocation of this “master regulator” of cellular antioxidant defenses is also induced by upregulated activity of SIRT-1 (Chai et al., 2018) and PPARγ co-activator 1-α (Cherry et al., 2014).

### Activation of PPARγ

The upregulation of PPARγ has been reported in the brains of study animals within a few days of the advent of a ketotic state (Grabacka et al., 2016b; Simeone et al., 2017b; Knowles et al., 2018). There are several mechanisms that could drive this phenomenon such as the activation of PGC-1α (Yun et al., 2018) and SIRT-1 (Fujita and Yamashita, 2018). However, the transcription factor is the prime regulator of ketogenesis and ketolysis and may be upregulated by the presence of high levels of fatty acids or KBs (Grabacka et al., 2016b; Simeone et al., 2017b; Knowles et al., 2018).

PPARγ may bind to genes and recruit transcriptional corepressor complexes that have the effect of repressing gene expression. Alternatively, it may bind to and/or sequestrate cofactors necessary for the activation of genes via a series of protein-protein interactions in a phenomenon described as transrepression (Tyagi et al., 2011; Polvani et al., 2012).

PPARγ activation has a well-documented role in reducing levels of inflammation in the brain and in the periphery by inhibiting the transcription of cytokines by sequestrating transcription factors such as activator protein 1 (AP-1), Signal transducer and activator of transcription 1 (STAT-1), and Nuclear factor of activated T-cells (NFAT), which positively regulate their expression (Yang et al., 2008; Tyagi et al., 2011). Perhaps unsurprisingly, increased activity of this nuclear hormone receptor also inhibits NF-κB–mediated inflammatory signaling via several mechanisms, which include the upregulation of NF kappa light polypeptide gene enhancer in B-cells inhibitor, alpha, a negative regulator of NF-κB (Scirpo et al., 2015) and acting as an E3 ligase, thereby increasing the proteosomal degradation of NF-κB p65 (Hou et al., 2012). PPARγ also appears to bind to the NF-κB p65 promoter region and directly suppresses the transcription of this subunit (Park et al., 2009; Remels et al., 2009). The various inhibitory effects of PPARγ on NF-κB are of particular interest as far as reducing neuroinflammation is concerned as they appear to be 1 element enabling PPAR to act as a major player in inducing antiinflammatory phenotypes in microglia (Cullingford, 2008; Jeong et al., 2011; Sikder et al., 2018).

PPARγ is also another major regulator of cellular redox status and acts at the junction of several signaling pathways and is involved in the upregulation of FOXO3a and Nrf-2 (Polvani et al., 2012). The upregulation of PPAR is associated with increased levels and activity of SOD, catalase, GPx, glutathione, UCP2, and Heme oxygenase-1 (HO-1) (Kim and Yang, 2013; Sekulic-Jablanovic et al., 2017). It could be argued that this antioxidant effect is exercised via stimulation of Nrf-2 and FOXO3a signaling, but PPAR/RXR dimers seem to have independent effects on upregulating several players in the cellular antioxidant defense network via binding to PPAR responsive elements of genes such as HO-1, Catalase (CAT), and SOD (Kim and Yang, 2013; Ndisang, 2014; Sekulic-Jablanovic et al., 2017).

PPARγ upregulation can also positively regulate oxidative metabolism by stimulating the transcription of genes governing rates of mitochondrial glucose metabolism and mitochondrial beta-oxidation of free fatty acids (Monsalve et al., 2013; Corona and Duchen, 2016). These activities are largely carried out via the activation of PGC-1α, and readers interested in the pathways involved are invited to consult the work of (Fan and Evans, 2015) and (Govindarajulu et al., 2018).

## Future Directions and Conclusions

Given the data discussed above, it is reasonable to conclude that a therapeutic intervention based on induced ketosis could potentially alleviate many of the elements known to be involved in the pathophysiology, and possibly the pathogenesis, of neuroprogressive illnesses. However, studies relating to the use of the approach in such illnesses are currently limited to very small prospective or retrospective open label studies or internet-based analysis of patient feedback (Bostock et al., 2017b; Brietzke et al., 2018), clearly indicating a need for well-designed and adequately powered randomized blinded controlled trails assessing efficacy and safety. We now move on to considering major issues that need to be taken into account while designing such studies, with the main focus being safety and compliance.

Generally, dietary-induced ketosis in rodents involves the use of commercial preparations at approximately 5.2 Kcal/g with 70% fat, 20% protein, and 10% carbohydrate. The sources of fat are usually Medium-chain triglycerides (MCTs) coupled with range of oils such as canola oil or flax seed oil. Casein is normally the sole source of protein source and maltodextrin usually serves as the sole source of carbohydrates (Hyatt et al., 2016). A classical or modified KD is usually started in hospital when children are concerned, but adults usually commence such diets in the community (Kossoff and Hartman, 2012). The use of KB supplements can largely avoid the side effects associated with the transition to ketosis often described in the grey literature as “keto flu.” The classical KD diet, with a very high intake of fatty foods, may produce a range of gastrointestinal side effects such as nausea, vomiting, dehydration, constipation, low appetite, and, most commonly, diarrhea (Włodarek, 2019). Other minor side effects include headache, muscle cramps, rashes, general weakness, and halitosis (Harvey et al., 2018). Unsurprisingly, the therapeutic utility of the unmodified KD may be limited by issues of poor tolerability and compliance from the perspective of patients and caregivers. In fact, a recent study based on the meta-analysis of 45 studies concluded that the compliance rate of children on a classical KD for the treatment of refractory epilepsy over a 2-year period was 29% (Cai et al., 2017). An earlier meta-analysis reported a somewhat higher but still troublesome compliance rate of 42% for adults over the same time period (Ye et al., 2015). This is comparable with the compliance rate of adults consuming a classical KD for the treatment of type 2 diabetes (Stern et al., 2004; Westman et al., 2008), which is obviously relevant from the perspective of treating patients with neuroprogressive disorders who will probably require long-term if not continuous consumption of this diet. The use of medium chain triglycerides to induce ketosis has allowed the ingestion of significantly lower levels of dietary fat compared with the 3 or 4:1 fat to carbohydrate and protein ratios used in the orthodox or classical KD, which has improved tolerability somewhat, although the high “dropout rates” observed in studies still remain primarily caused by unpleasant gastric side effects (Harvey et al., 2018; Taylor et al., 2018).

There are also concerns among many clinicians regarding the safety of a high-lipid diet due to the potential of increasing total Low-density lipoprotein (LDL), and Very low-density lipoprotein (VLDL) levels, potentially increasing the patient’s risk of developing obesity, insulin resistance, metabolic syndrome, Type 2 diabetes (T2D), and cardiovascular disease (Kosinski and Jornayvaz, 2017; Bolla et al., 2019). Indeed, there is evidence that the use of the classical KD may induce dyslipidemia, at least in the short term (Kossoff et al., 2018), and that this phenomenon may also apply to individuals on a MCT-based KD as there is a report of increased LDL and total cholesterol in healthy volunteers consuming such a diet (Tholstrup et al., 2004). This is particularly relevant as far as patients suffering from neuroprogressive disorders are concerned as there is a wealth of evidence showing an increased prevalence of metabolic abnormalities and cardiovascular disease in such individuals compared with age and sex norms even before the instigation of any therapeutic interventions (for review, see Morris et al., 2019c). Clearly, patient safety is paramount, and hence we will examine evidence regarding the effects of prolonged induced ketosis before examining ways of improving patient tolerance.

There is now a large and accumulating body of evidence associating short-term prolonged ingestion of a KD with significant weight loss in obese adults, which is accompanied by a decrease in systemic inflammation, reduced insulin resistance, and, on balance, an increased capacity for exercise (Paoli, 2014; Paoli et al., 2015; Hall and Chung, 2018; Bolla et al., 2019; for review, see Murphy and Jenkins, 2019). There are also several studies reporting favorable effects on metabolic syndrome, including evidence of reversal (Staverosky, 2016; Gibas and Gibas, 2017; Gershuni et al., 2018). Remarkably, there is evidence to suggest that the improvement in all parameters of metabolic syndrome in patients ingesting an MCT-based KD may be greater than in patients consuming a low-fat diet combined with engaging in rigorous exercise regimes (Gibas and Gibas, 2017). Perhaps unsurprisingly, given the data discussed thus far, there is also an accumulating body of evidence associating the short-term or prolonged consumption of a MCT-based or classical KD with a clinically significant decline in HbA1c and improvements in glycemic control in patients with T2D (Westman et al., 2008; Hussain et al., 2012; Krebs et al., 2013; Goday et al., 2016). In addition, there are a number of studies reporting a significantly reduced need for medication in T2D patients on a KD compared with patients on high-carbohydrate or low-fat diets (for review, see Westman et al., 2008).

However, consideration of the data provided by some of these studies suggests that adults with metabolic abnormalities should be careful to avoid long chain triglycerides and/or saturated fats as a vehicle to induce ketosis as this may result in elevated levels of LDL (Westman et al., 2008; Brinkworth et al., 2009). This is consistent with data obtained from studies involving children with pharmaceutically resistant epilepsy where researchers have reported significant increases in LDL, VLDL, and total cholesterol in their study participants (Kwiterovich, 2003; Groesbeck et al., 2006; Nizamuddin et al., 2008; Güzel et al., 2016; Zamani et al., 2016). However, in most cases, these increases appear to be short term and transitory and normalize within 12 months (Kwiterovich, 2003; Groesbeck et al., 2006; Liu et al., 2013; Kapetanakis et al., 2014). This would also appear to be the case for individuals with preexisting hyperlipidemia prior to the commencement of the classical KD (Liu et al., 2013). In addition, there is evidence that children who have been on the classical KD for 6 years display no evidence of hyperlipidemia (Groesbeck et al., 2006).

From the perspective of chronic administration in adult patients with neuroprogressive illnesses, it also seems reassuring to note that even in a scenario where the use of a classical KD leads to increases in LDL cholesterol in patients with T2D, there would appear to be an even greater relative increase in HDL cholesterol and reduction in levels of triglycerides, greater increase in HDL cholesterol, and a decrease in serum triglycerides (Westman et al., 2008; Brinkworth et al., 2009). Other authors have also reported increases in HDL levels (Sharman et al., 2002; Foster et al., 2003; Samaha et al., 2003; Volek et al., 2005; Dashti et al., 2006; Brinkworth et al., 2009; Tay et al., 2014) following prolonged diet-induced ketosis in human studies and reductions in triglyceride in conjunction with increased levels of HDL (Foster et al., 2003; Samaha et al., 2003; Dashti et al., 2006; Brinkworth et al., 2009; Tay et al., 2014). For the sake of completeness, it should also be noted that a solitary study has reported a reduction in LDL cholesterol in patients consuming a MCT-based KD (Dashti et al., 2006).

Further reassurance may be obtained by the results of a recent meta-analysis of 13 randomized controlled studies that concluded that MCT ingestion has at minimum no adverse effects on the lipid profiles of humans in the short or longer term (Mumme and Stonehouse, 2015). It is also noteworthy that the addition of MCTs to a classical KD diet is often used in clinical practice to ameliorate dyslipidemia induced by a classical KD (for review, see Kossoff et al., 2018). There is also a growing body of evidence to suggest that the beneficial effects of MCTs are at least equal to those provided by extra virgin olive oil (St-Onge et al., 2008; Namayandeh et al., 2013; Chinwong et al., 2017; Khaw et al., 2018). Readers interested in the evidence relating to the beneficial effects of olive oil on human lipid profiles are referred to an excellent treatment of the subject by (Høstmark and Haug, 2013). There is also evidence to suggest that that caprylic to capric ratio in MCT preparations is an important element underpinning their favorable or neutral effect on lipid profiles, with high levels of caprylic acid being particularly important (for review, see Bach et al., 1996). It is also reassuring to note that use of modified tricaprylin and other MCT-based KDs has also produced encouraging results in the treatment of Alzheimer’s disease, with significant (albeit modest) improvements in memory scores and overall cognitive functions in patients with mild or moderate disease without any significant adverse effects on any metabolic parameters (Reger et al., 2004; Henderson et al., 2009; Taylor et al., 2018; Ota et al., 2019). Similar results have also been achieved via the use of MCT-based KDs in patients with mild cognitive impairment (Krikorian et al., 2012).

There may be an opportunity to further reduce the amounts of MCT oil consumed each day, thereby minimizing gastrointestinal side effects and aiding compliance, by using caprylic MCTs as these molecules are about 3 times more ketogenic than their capric equivalents and upwards of 6 times more ketogenic than C12 MCTs, thereby offering the prospect of therapeutic levels of BHB from reduced amounts of MCT oil ingested (St-Pierre et al., 2019). Side effects may also be minimized via the emulsification of MCT oils before ingestion, which also offers the prospect of ketosis achieved by lower daily amounts of these oils as emulsified MCTs may increase plasma ketones twofold compared with an equivalent amount of nonemulsified MCT oils (Courchesne-Loyer et al., 2017b). In addition, there are also data to suggest that the use of emulsified MCT oil as the means of inducing ketosis can reduce overall side effects by some 50% compared with an equivalent amount of nonesterified oil (Courchesne-Loyer et al., 2017b). However, despite such approaches, poor compliance remains a major issue with classical and MCT-based KDs, and it is also fair to say that long-term prospective studies to assess the effects of induced ketosis over several years of continuous consumption have not yet been carried out (Kossoff et al., 2018; Murphy and Jenkins, 2019).

These enduring problems with tolerability and lingering concerns over the development of dyslipidemia in long-term use have led to an increasing research focus on the use of exogenous ketone supplements in the form of KB salts and esters, with common examples being 1, 3-butanediol monoester of BHB and glyceryl-tris-3-hydroxybutyrate, which provide high levels of BHB directly to the body without the need to induce ketogenesis and thus without elevations in free fatty acids and, perhaps more importantly thus far, no evidence of induced dyslipidemia (Hashim and VanItallie, 2014; Veech, 2014). In fact, there is accumulating evidence to suggest that orally administered KB esters or salts result in the inhibition of adipocyte lipolysis (Evans et al., 2017; for review, see Pinckaers et al., 2017) and the inhibition of cholesterol synthesis (Kemper et al., 2015).

The research focus on KB supplementation is not just concerned with improving safety; however, there is accumulating evidence that the administration of R-1, 3-butanediol from ketone monoesters can produce plasma levels of KBs in humans that are at least as high as those produced by the most rigorous consumption of the classical KD (Clarke et al., 2012; Hashim and VanItallie, 2014; Stubbs et al., 2017). For example, at a single dose of 395 mg/kg, KB supplementation in human adults can increase levels of BHB in the plasma to 3.3 mmol/L whether administered as a capsule or in a drink (Clarke et al., 2012; Stubbs et al., 2017). In addition, there are data to suggest that the administration of ketone esters or salts as drinks over a 24-hour period can deliver 24 g of BHB as effectively as an infusion (Stubbs et al., 2017).

There is also a suggestion that ketone ester or salt drinks can be modified to produce a relatively low level of KB supplementation and increase the ratio of carbohydrates and proteins in the mix (Choi et al., 2018). This method has produced therapeutic levels of BHB following drinks containing a KB ester to carbohydrate to protein ratio of 1:7:1, which approaches the composition of a standard diet and may further improve tolerability and compliance (Choi et al., 2018). The use of ketone ester drinks also induces levels of BHB in plasma, which does not appear to be significantly affected by food intake, and thus the use of KB drinks could avoid the rigors of dietary restriction. This would appear to be another potential benefit in terms of a long-term therapeutic intervention for patients with neuroprogressive disorders (Stubbs et al., 2017; Kovács et al., 2019).

In this comprehensive review, it has been suggested that induced ketosis or protracted KB ingestion may reduce oxidative stress, improve cellular bioenergetics, and upregulate the activity of PPAR, SIRT-1, and AMPK as well as brain NAD^+^ levels. These changes speak to the potential therapeutic value of this dietary change for neuroprogressive disorders such as SZ, BPD, and MDD and suggest that clinical trials of ketogenic dietary strategies focusing on the use of ketone esters in these disorders are timely.
